# A non-synonymous coding change in the *CYP19A1 *gene Arg264Cys (rs700519) does not affect circulating estradiol, bone structure or fracture

**DOI:** 10.1186/1471-2350-12-165

**Published:** 2011-12-20

**Authors:** Jenny Z Wang, Mandeep S Deogan, Joshua R Lewis, Shelby Chew, Ben H Mullin, Tegan J McNab, Scott G Wilson, Evan Ingley, Richard L Prince

**Affiliations:** 1 School of Medicine and Pharmacology, University of Western Australia, Perth, WA, Australia; 2 Department of Endocrinology and Diabetes, Sir Charles Gairdner Hospital, Nedlands, WA, Australia; 3 School of Biological Sciences, Murdoch University, Murdoch, WA, Australia; 4 Cell Signalling Group, Western Australian Institute for Medical Research and Centre for Medical Research, University of Western Australia, Perth, WA, Australia

## Abstract

**Background:**

The biosynthesis of estrogens from androgens is catalyzed by aromatase P450 enzyme, coded by the *CYP19A1 *gene on chromosome 15q21.2. Genetic variation within the *CYP19A1 *gene sequence has been shown to alter the function of the enzyme. The aim of this study is to investigate whether a non-synonymous Arg264Cys (rs700519) single nucleotide polymorphism (SNP) is associated with altered levels of circulating estradiol, areal bone mineral density or fracture.

**Methods:**

This population- based study of 1,022 elderly Caucasian women (mean age 74.95 ± 2.60 years) was genotyped for the rs700519 SNP were analyzed to detect any association with endocrine and bone phenotypes.

**Results:**

The genotype frequencies were 997 wildtype (97.6%), 24 heterozygous (2.3%) and 1 homozygous (0.1%). When individuals were grouped by genotype, there was no association between the polymorphism and serum estradiol (wildtype 27.5 ± 16.0; variants 31.2 ± 18.4, P = 0.27). There was also no association seen on hip bone mineral density (wildtype 0.81 ± 0.12; 0.84 ± 0.14 for variants, P = 0.48) or femoral neck bone mineral density (0.69 ± 0.10 for wildtype; 0.70 ± 0.12 for variants, P = 0.54) before or after correction of the data with age, height, weight and calcium therapy. There were also no associations with quantitative ultrasound measures of bone structure (broadband ultrasound attenuation, speed of sound and average stiffness).

**Conclusions:**

In a cohort of 1,022 elderly Western Australian women, the presence of Arg264Cys (rs700519) polymorphism was not found to be associated with serum estradiol, bone structure or phenotypes.

## Background

Aromatase P450 enzyme (*CYP19A1) *is located in the endoplasmic reticulum of cells that synthesize estrogen, it catalyses the conversion of C_19 _steroidal androgens, testosterone, androstenedione and hydroxyl androstenedione into C_18 _steroidal estrogens estradiol, estrone and estriol, respectively. Bone structure, as measured by dual energy-x-ray absorptiometry (DXA) areal bone mineral density (aBMD) is a phenotype dependent on circulating estradiol in postmenopausal women [1]. Another non-invasive modality of bone structural assessment, heel quantitative ultrasound and its modalities (including broadband ultrasound attenuation (BUA) speed of sound (SOS) and average stiffness) are also related to estradiol production [2].

The aromatase enzyme is encoded for by the *CYP19A1 *gene which is localized on 15q21.2 and spans approximately 131 Kb. Individual genetic variations in the aromatase sequence have previously been described that have substantial phenotypic effects. For example a homozygous single base change at bp 1123 (C→T) can lead to severe generalized aromatase deficiency [3] while a TTTA repeat polymorphism has also been shown to be associated with circulating estrogen, bone structure, and biochemistry in older women [4].

Four non-synonymous coding SNPs, Trp39Arg, Met364Thr, Thr201Met and Arg264Cys, have been studied to date [5]. Trp39Arg is a rare polymorphism associated with breast cancer [6]. Met364Thr, observed only in the Han Chinese-American samples, is related to a significant decrease in affinities for the androgen substrate [7] whilst the Thr201Met variant is related to an increase in the aromatase enzyme activity [8]. In this study, associations between the sequence variant rs700519 (Arg264Cys) in exon 7 of the *CYP19A1 *gene, estradiol and bone phenotypes were examined.

## Methods

### Subjects

The rs700519 SNP was genotyped in 1,022 individuals who participated in the Calcium Intake Fracture Outcome Study (CAIFOS), a 5-year randomised controlled trial looking into the effects of calcium supplementation in prevention of osteoporotic fractures [9]. The subjects were all Caucasian women, aged 70 to 85 years and represented a random population sample of this age group. No participant was retained in the study if they were taking any medication that would have a known effect on bone metabolism or were suffering from a disease that suggested the subject would be unlikely to survive for 5 years. Subjects received either 1.2 g of elemental calcium in the form of calcium carbonate or placebo daily. At baseline all participants completed a lifestyle questionnaire and had their height and weight measured. The study was approved by Human Research Ethics Committee office of Sir Charles Gairdner Hospital, Western Australia. All participants gave informed consent for the study.

### Areal bone mineral density and quantitative ultrasound measurements

At baseline, quantitative ultrasound (QUS) of the left calcaneus was measured twice in all subjects. Measurements were taken using Lunar Achilles ultrasound machine (Lunar Corp, USA) employing the manufacturer's quality assurance methods. The average of the two measurements of speed-of-sound (SOS), broadband ultrasound attenuation (BUA) and stiffness were recorded and used in the analyses. Using the manufacturer's standards, the coefficients of variation (CV) for SOS and BUA were 0.43% and 1.59% respectively [1].

Areal bone mineral density (aBMD) was measured using dual-energy X-ray absorptiometry (DXA) on a Hologic 4500A machine (Hologic, Boston, MA, USA) at the total hip and femoral neck regions at year 1 of the study. The CV at the total hip and femoral neck were 1.0% and 1.4% respectively [1].

### Fracture status

Clinical incident fracture due to a minimal trauma, as defined by falling from a height of less than 1 metre, was assessed during the 84 month follow-up using radiographic reports. Fractures of the face, skull, fingers and toes were not included.

### Biochemistry

At baseline a blood sample from each subject was collected following an overnight fast. Serum estradiol was measured using a radioimmunoassay (Orion Diagnostica, Finland) inter-assay CV 5.0% at 18 pmol/L and 6.6% at 100 pmol/L, intra-assay CV 5.0% at 100 pmol/L). Sex hormone-binding globulin (SHBG) was measured using an immunochemiluminometric assay (Imulite Co., LA, USA) the inter- and intra-assay CVs were 6.8% and 7.1%, respectively at 24 nmol/L. The free estradiol index (FEI) was calculated as the molar ratio of estradiol to SHBG. Genomic DNA was extracted and purified from ethylenediamine tetra-acetic acid (EDTA)-prepared peripheral blood for genotyping analyses [1].

### rs700519 SNP genotyping

rs700519 SNP in the *CYP19A1 *gene was analysed using a Restriction Fragment Length Polymorphism (RFLP) method. In brief, the PCR reaction consisted of: 1x PCR buffer, 0.2 mM dNTP mix (Promega, USA), 0.5 mM MgCl_2 _solution, 1.5 uM of each of each sequence specific forward (5' GGCAAATAAATCTGTTTCGCTAGA 3') and reverse (5' CAACTCAGTGGCAAAGTCCA 3') primer (Sigma Aldrich, Australia), 0.5 U of HotStarTaq *Plus *DNA polymerase and 20 ng of DNA made up to a final reaction volume of 20 μl using PCR grade H_2_O. The PCR cycling conditions were 95°C for 5 min, 35 cycles of 94°C for 1 min, 68°C for 1 min and 72°C for 1 min followed by a final extension at 72°C for 10 min. The PCR product was digested with 1 U of the restriction enzyme HpyCH4V at 37°C for 2 hours and the product analysed by electrophoresis on an agarose gel (2%). A positive control was included in each reaction.

In these analyses, individuals who had the rs700519 wildtype genotype displayed a 173 bp amplicon, while those heterozygous had bands at 173 bp, 118 bp and 55 bp and individuals who were homozygous for the rare variant, revealed bands at 118 bp and 55 bp.

### Data and statistical analysis

Statistical analysis was performed using SPSS (SPSS Inc., Chicago, IL, USA, version 16). For association testing a dominant model was used. The differences in phenotypic means for each genotype group were examined using analysis of covariance (ANCOVA), with the covariates age, height, weight and calcium therapy incorporated in the analysis of BMD and QUS; while age and calcium therapy were incorporated of biochemical phenotypes. The fracture phenotypes were compared by Cox regression adjusted for age, weight, height and calcium therapy. A post hoc power calculation was undertaken to estimate the probability of a Type 2 error occurring in the study [10].

## Results

### Characteristics of study population

The demographics, bone density, QUS and biochemistry data for the CAIFOS population are presented in Table 1.

**Table 1:** Demographics, bone density, quantitative ultrasound, and bone biochemistry of the CAIFOS population (n = 1022)

Variable	Mean ± SD
**Age (years)**	74.9 ± 2.6
**Weight (Kg) **	68.4 ± 11.9
**Height (cm) **	159.0 ± 5.8
**BMI (kg/m^2^) **	27.1 ± 4.5
**Total hip BMD (mg/cm^2^) **	0.8 ± 0.1
**Femoral neck BMD (mg/cm^2^) **	0.7 ± 0.1
**Total fracture in 84 months (yes/no)**	205 (20.1%)
**Hip fracture in 84 months (yes/no)**	29 (2.8%)
**Vertebral fracture in 84 months (yes/no)**	71 (6.9%)
**Estradiol (pmol/L) **	27.6 ± 16.1
**SHBG (nmol/L) **	54.9 ± 23.7
**Free estradiol index (units)**	2.9 ± 3.0
**Average BUA (dB/MHz) **	100.4 ± 7.6
**Average SOS (m/s) **	1512.6 ± 24.9
**Average stiffness (%) **	70. 5 ± 11.0

Results in mean ± SD or number (percentage).

### Association between SNP rs700519, estradiol and bone phenotypes

The genotype frequencies were 997 wildtype (97.6%), 24 heterozygous (2.3%) and 1 homozygous (0.1%) for the variant. Age, height, weight and BMI were not significantly different between the groups. There were no associations between genotype and circulating estradiol, SHBG and FEI. For bone phenotypes there were no associations between aBMD before and after adjusted for age, height, weight and calcium therapy both for total hip and femoral neck sites or ultrasound bone phenotypes including BUA, SOS and average stiffness. No association was evident between genotypes and total, hip or vertebral fracture incidence over 84 months (Table 2).

**Table 2:** BMS, QUS and sex hormone biochemistry parameters in relation to the distribution of the rs700519 genotype in CAIFOS study (n = 1022)

Variable	Wild type	Variants	*P *value
**Number**	997	25	
**Age (years)**	74.95 ± 2.60	74.91 ± 2.76	0.83
**Weight (Kg) **	68.32 ± 11.79	72.19 ± 15.85	0.11
**Height (cm) **	158.96 ± 5.86	159.35 ± 4.67	0.74
**BMI (kg/m^2^) **	27.03 ± 4.39	28.53 ± 6.81	0.10
**Total fractures (yes/no)**	198 (19.9%)	7 (28.0%)	0.27
**Hip fractures (yes/no)**	28 (2.8%)	1 (4.0%)	0.76
**Vertebral fractures n (yes/no)**	69 (6.9%)	2 (8.0%)	0.83
**Total hip BMD (mg/cm^2^) **	0.81 ± 0.12	0.84 ± 0.14	0.48
**Femoral neck BMD (mg/cm^2^) **	0.69 ± 0.10	0.70 ± 0.12	0.54
**Average BUA (dB/MHz) **	100.45 ± 7.61	99.46 ± 8.90	0.21
**Average SOS (m/s) **	1512.62 ± 24.66	1512.83 ± 33.87	0.90
**Average stiffness (%) **	70.52 ± 10.89	69.83 ± 14.90	0.50
**Estradiol (pmol/L) **	27.51 ± 16.00	31.16 ± 18.36	0.27
**SHBG (nmol/L) **	55.03 ± 23.81	50.52 ± 19.73	0.33
**Free estradiol index (units)**	2.95 ± 3.01	2.50 ± 2.10	0.43

Results in mean ± SD or number (percentage). *P *value generated using ANCOVA test or COX regression.

Assuming a QTL variance of 1% with a marker frequency of 0.013, where this marker is hypothesized to be the disease variant, at an alpha of 0.05 the sample size of 1,022 had statistical power of 0.83.

## Discussion

The Arg264Cys polymorphism causes a non-synonymous amino acid substitution which was not associated with bone or estradiol phenotypes in this cohort of elderly Caucasian women. Our study had sufficient statistical power to detect even relatively small effects and so the lack of association is unlikely to be due to a type II error.

This lack of effect on endocrine and bone metabolism phenotypes is generally supported by several previous studies of the Arg264Cys polymorphism in relation to breast cancer. Watanabe et al. reported that in Japanese women, the polymorphism had no relationship to the risk of breast cancer and did not affect an *in vitro *assay of aromatase activity using test using the tritiated water release assay in transfected COS-7 cells [11]. A case-control study in Austria reported that this polymorphism did not affect breast cancer or fibroadenoma risk [12] while in an *in vitro *study focusing on aromatase activity in breast cancer tissue, an Arg264Cys heterozygote was found but showed no alteration of aromatase activity [13].

In contrast, Ma *et al*. undertook studies using tritiated water release assays in, COS-1, cells transfected with cDNA containing the wild type and variant sequence of rs700519 [5]. The K_m _value of wide-type was reported to be 6.7 ± 2.0 nmol/L as compared to Arg264Cys 5.9 ± 2.6 nmol/L (mean ± SE, *P = *0.0001). However this group has described the presence of a double mutant with both Arg39 and Arg264Cys which may have affected the interpretation of their results. Thus it remains possible that the assay used by Ma et al does not reflect estradiol production in the adipocytes of postmenopausal women, the major source of circulating estradiol in the women studied in this paper. Another study, in a group of 1,136 Chinese breast cancer patients [14], reported that rs700519 was associated with breast cancer disease-free survival with an age-adjusted hazards ratio for the homozygote (Cys/Cys) of 2.2 (95%, CI 1.2-4.1), but did not report serum estradiol levels.

Ethnic groups show substantial differences in the allele frequency of Arg264Cys, estimates suggest that the Japanese population has a minor allele frequency (MAF) of 27.3%, in Chinese the MAF is 11.6%, for sub Saharan Africans 21.4% and European populations, 3.1% [15]. Our study shows a MAF of 1.3%, which was similar to the study in UK showing low frequency of Arg264Cys in British people [16].

## Conclusions

In conclusion this *CYP19A1 *gene Arg264Cys (rs700519) polymorphism has no effect on circulating estradiol or bone phenotypes in elderly Caucasian women. Whether it is possible that this SNP has an effect on breast cancer epidemiology or hormone metabolism in premenopausal women remains uncertain and will need to be determined from further study.

## Competing interests

The authors declare that they have no competing interests.

## Authors' contributions

Authors Obtained funding included RLP, SGW and EI; Authors participated in conception and design were MSD,RLP,SGW, JRL, BHM, SWC and EI; Authors carried out data analysis and interpretation included MSD, JZW, RLP, SGW, JRL, TJM and SWC; Authors who did drafting of the manuscript and critical revision were MSD, JZW, RLP, SGW, JRL, TJM. All authors have read and approved the final manuscript.

## Pre-publication history

The pre-publication history for this paper can be accessed here:

http://www.biomedcentral.com/1471-2350/12/165/prepub
